# Exercise and dietary program-induced weight reduction is associated with cognitive function among obese adolescents: a longitudinal study

**DOI:** 10.7717/peerj.3286

**Published:** 2017-05-16

**Authors:** Chun Xie, Xiaochun Wang, Chenglin Zhou, Chang Xu, Yu-Kai Chang

**Affiliations:** 1 School of Kinesiology, Shanghai University of Sport, Shanghai, Shanghai, China; 2 Graduate Institute of Athletics and Coaching Science, National Taiwan Sport University, Guishan Township, Taoyuan County, Taiwan

**Keywords:** Body mass index, Diet, Executive function, Physical activity

## Abstract

**Objective:**

The present study was to determine the effect of a combined exercise and dietary program on cognitive function as well as the relationship between the program-induced weight change and cognitive function alterations.

**Design:**

The study applies a quasi-experimental design.

**Methods:**

Fifty-eight adolescents with obese status (body mass index, BMI >28 kg/m^2^) were assigned to either an experiment (*n* = 30) or control group (*n* = 28). Participants in the experiment group received a scheduled program with a specific exercise protocol (two sessions per day, six days per week) and diet plan for four consecutive weeks; the control group was instructed to maintain their normal school activities. The primary outcome measures were anthropometric data and flanker task performance.

**Results:**

The combined program led to reduced BMI with maintenance of the incongruent accuracy in the experiment group, but the incongruent accuracy decreased in the control group after the four-week period. Additionally, the change in weight status between post- and pre-test measurements was inversely correlated with the change in incongruent accuracy.

**Conclusion:**

The combined exercise and dietary program resulted in decreased weight and enhanced executive function in the obese adolescents, and the weight alteration may be considered the mediator between the intervention and executive function.

## Introduction

Obesity in children and adolescents is a crucial health concern due to its epidemic proportions globally, with 17% of the 2- to 19-year-old population in the United States (Ogden et al., 2016) and approximately 15% of the pediatric population in China being classified as overweight and obese (Ji, Chen & China WGoO, 2013). According to the Global Burden of Disease Study 2013, the prevalence of obesity in children and adolescents has changed substantially in both developed and developing countries (Ng et al., 2014). Obesity is likely to continue from childhood and adolescence to adulthood (World Health Organization, 2016) and is associated with an increased risk of premature mortality and a variety of adverse health outcomes, including diabetes, hypertension, ischemic heart disease, stroke, disability, and asthma (Reilly & Kelly, 2011).

Obesity-related negative outcomes are not merely physical but also mental, and an obesity-associated decline in cognitive function has been proposed. A systematic review that focused on whether obesity was associated with cognitive impairment found that obesity was inversely correlated with several cognitive domains, such as complex attention, verbal and visual memory, and decision making (Prickett, Brennan & Stolwyk, 2015). Similar lower performances were also observed in executive function, which is a higher or meta level of cognitive function, in obese children relative to their normal weight counterparts (Reinert, Po’e & Barkin, 2013). These cognitive dysfunctions in obesity may be attributed to altered brain structures and/or deficits in the orbitofrontal cortices (Reinert, Po’e & Barkin, 2013), hippocampus, white matter, mid-posterior corpus callosum, and globus pallidus volumes (Bauer et al., 2015). Because these cognitive functions are vulnerable to alteration during childhood, a crucial developmental stage, the identification of interventions that ameliorate obesity and its associated negative effects is urgent for the young population.

Both exercise and diet have independently been recognized as effective approaches for treating weight loss (Donnelly et al., 2009). Interestingly, exercise and diet separately affect cognitive function. For example, an early meta-analytic review reported a significant positive effect of exercise on cognitive function in children, with an overall effect size of 0.32 (Sibley & Etnier, 2003). Recently, more daily physical activity was correlated with better executive function in primary school-aged children, wherein sedentary behavior was related to poorer executive function. Randomized controlled trials have also demonstrated a similar beneficial effect: for young individuals, an exercise program that ranged from three to nine months not only improved the behavioral performances in tasks associated with executive function (Chen et al., 2016) but also led to positive alterations in brain activation (Chaddock-Heyman et al., 2013; Hillman et al., 2014; Krafft et al., 2014) compared to those for the control intervention, suggesting a causal effect of exercise on cognitive functions in the young population.

The relationship between diet and cognitive function was indirectly established. For example, a high-fat diet is linked to a deficiency in hippocampal-dependent memory (Beilharz, Maniam & Morris, 2015; Kanoski & Davidson, 2011), as well as cognitive decline and dementia (Freeman et al., 2014), and the underlying mechanism of the dysfunction is postulated to be poor-diet-induced insulin resistance, inflammation and oxidative stress (Freeman et al., 2014). In contrast, compared to their counterparts in the control group, healthy elders who had long-term diet restriction consisting of a 30% reduction in calories experienced improved memory (Witte et al., 2009); similarly, calorie restriction improved the working memory of male mouse lemurs (Dal-Pan et al., 2011). Although studies associated with diet and cognitive function in children are limited, poor diet quality has been linked to lower cognitive performance in the other populations (Haapala et al., 2015). These findings provide initial evidence of the effects of diet restriction on specific cognitive functions.

Notably, the combination of exercise and diet may provide a larger effect than each single intervention alone. For example, a program that combined exercise and diet offered more long-term weight loss (Johns et al., 2014; Wu et al., 2009) or an improved metabolic profile (e.g., high-density lipoprotein cholesterol and fasting glucose) (Ho et al., 2013) than a diet-only program. Indeed, policies using a school diet or physical activity alone insufficiently prevented obesity in children, and a program involving a multifaceted approach is required (Williams et al., 2013). These findings suggest that it would be advantageous to examine combined interventions utilizing exercise and diet. To our knowledge, only one study has examined the effects of a multifaceted intervention on cognition in the obese population. The study focused on overweight adults with high blood pressure; specifically, Smith et al. (2010) observed that interventions involving both exercise and diet restriction exhibited superior performance in terms of both executive function and psychomotor speed, wherein an intervention with diet alone only improved the psychomotor speed. However, whether the beneficial effect of the combined intervention extends to the young obese population remains undetermined.

The purpose of the present study was to examine the effect of a combined exercise and dietary program on the cognitive function of obese adolescents. Additionally, whether the combined-program-induced weight change was associated with cognitive function was determined.

## Methods

### Study design and participants

The study is a quasi-experimental design, with the group defined as the between-subject factor and the time point as the within-subject factor. Fifty-eight adolescents with ages between 14 and 17 years were recruited from the Yangpu district of Shanghai. Participants who met the following initial eligibility requirements were included: (a) obesity status, as assessed by body mass index (BMI) >28 kg/m^2^ (the cutoff point was based on the weight categories in Asia) (Barba et al., 2004); (b) absence of endocrine and cardiovascular diseases; (c) absence of neurological disorders or major disease; (d) no consumption of pills that could affect glucose metabolism, lipid metabolism, or diet; (e) ability to safely conduct the exercise regimen, as assessed by the Physical Activity Readiness Questionnaire; (f) normal or corrected-to-normal vision; and (g) right-hand dominance.

Eligible participants from the Shanghai Dianfeng Fat Loss Camp (a weight loss camp) located at the Shanghai University of Sport were chosen as the experiment group (*n* = 30); participants from a senior high school affiliated with the University of Shanghai for Science and Technology were chosen as the control group (*n* = 28). Table 1 summarizes the demographic background regarding gender, age, height, weight, as well as amount of physical activity assessed by short form International Physical Activity Questionnaire (IPAQ-SF) (Craig et al., 2003) of the two groups. Both the participants and their parents/guardians provided written informed consent that was in accordance with the ethical guidelines of the Declaration of Helsinki, and the study was approved by the Institutional Review Board of the Shanghai University of Sport (#2015030).

**Table 1:** Description of the demographic background of the two groups.

Variable	Experiment group (*n* = 30)	Control group (*n* = 28)	Total (*n* = 58)
Gender (Female)	8	10	18
Age (years)	15.07 ± 0.83	15.18 ± 0.39	15.12 ± 0.65
Height (cm)	169.67 ± 8.98	169.21 ± 7.25	169.45 ± 8.13
Weight (kg)			
Pre-test	95.23 ± 17.56	88.73 ± 10.10	92.09 ± 14.69
Post-test	84.98 ± 16.26	87.48 ± 10.20	86.19 ± 13.62
Change	−10.25 ± 2.52^**^	−1.25 ± 2.69	−5.90 ± 5.22
BMI (kg/m^2^)			
Pre-test	32.83 ± 3.84^*^	30.90 ± 1.95	31.90 ± 3.20
Post-test	29.19 ± 3.52	30.47 ± 2.13	29.81 ± 2.98
Change	−3.64 ± 0.78^**^	−0.43 ± 0.88	−2.09 ± 1.81
Physical activity (METs)			
Vigorous	1150.93 ± 1335.32	1231.43 ± 967.28	1191.18 ± 1151.30
Moderate	1179.33 ± 993.64	902.86 ± 661.31	1041.10 ± 827.48
Low	1000.37 ± 1061.29	853.89 ± 942.68	927.13 ± 1001.99
Sedentary	878.33 ± 870.62	714.64 ± 162.90	796.49 ± 516.76
Total	3826.63 ± 1649.68	3702.82 ± 1483.65	3764.73 ± 1566.67
Basal metabolic rate (kJ/m^2^/h)	1898.37 ± 268.30	1814.97 ± 209.32	1856.67 ± 238.81

**Notes.**

* *p* < 0.05.

** *p* < 0.01.

BMI body mass index Change Difference between post-test and pre-test MET Metabolic Equivalent

**Figure 1:**
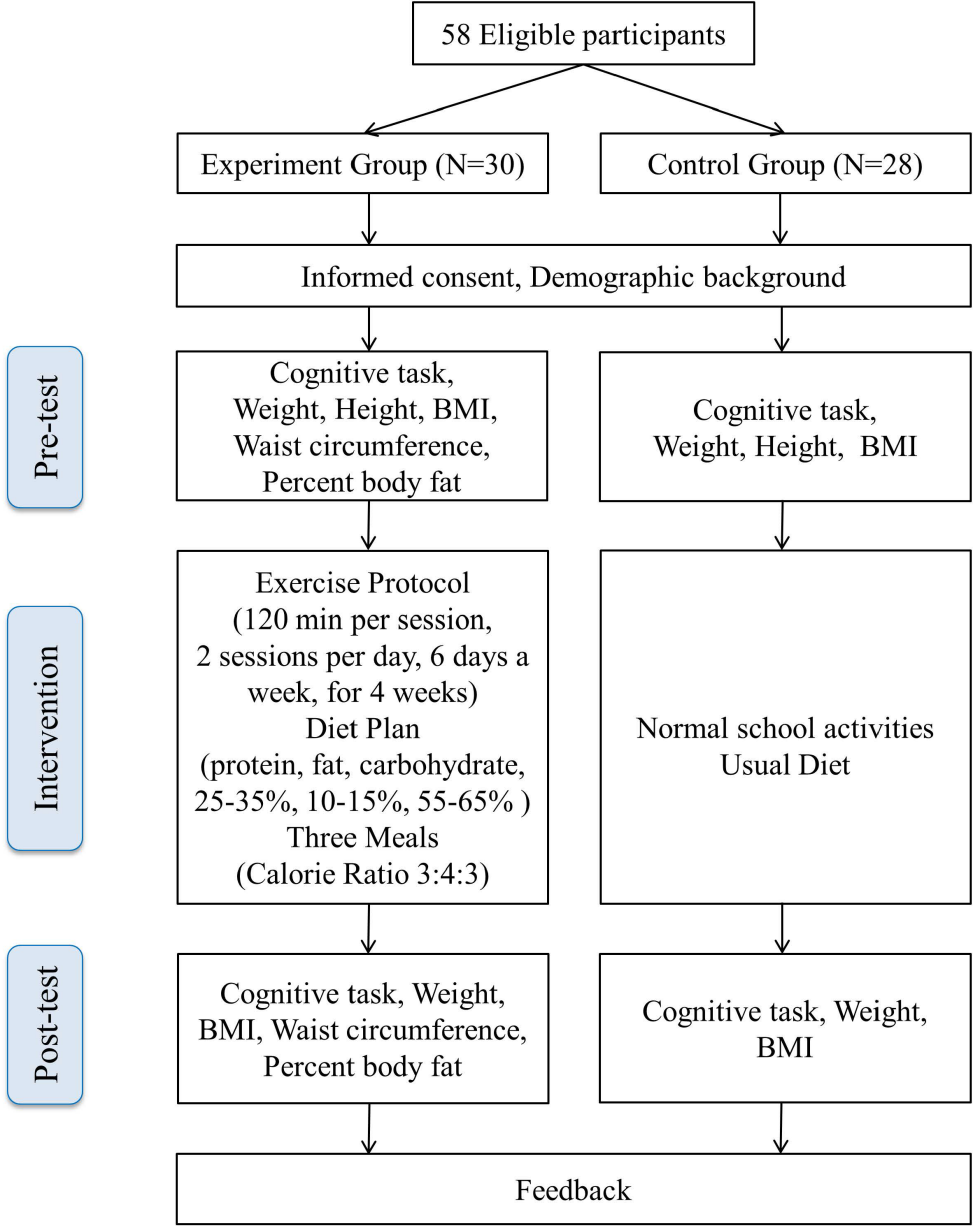
Flow chart of the experimental procedure.

Eligible participants in both groups had their demographic information, anthropometric data, and cognitive function measures assessed by a trained experimenter as the pre-test data. Participants in the experiment group underwent the four-week exercise and dietary program described below; the participants in the control group acted as wait-list control, with instructions to maintain their normal school activities and usual diet behavior. Following the intervention, anthropometric data and cognitive function measures were collected again as post-test data. The participants were informed the purpose and results of the study and provided feedback following analysis of the post-test data (Fig. 1).

### Exercise and dietary program

A combined exercise and dietary program, provided by the Shanghai Dianfeng Fat Loss Camp, was employed for participants in the experiment group. The camp consisted of a four-week program that aimed for weight reduction in obese adolescents. The camp participants received a scheduled routine daily, including the times to wake up and sleep, three meals, and homework, and they also followed a specific exercise protocol and diet plan. This protocol was effective in reducing the anthropometric parameter, serum lipid levels, and glycemic-related factors in overweight adolescents (Wang, Chen & Chen, 2011).

#### Exercise protocol

The exercise protocol comprised two sessions per day (i.e., morning and afternoon courses), six days per week for four consecutive weeks (48 sessions in total). Each session lasted 120 min and comprised three stages of (a) 18 min warm-up; (b) 84 min low- to moderate-intensity exercise with a target heart rate zone within 110–130 bpm; and (c) 18 min cool-down. A variety of exercise modes were provided (e.g., treadmill walking, swimming, badminton, and basketball) to create an interesting exercise environment. During exercise, two instructors monitored the heart rate by polar heart rate monitor that was attached to each participant. The heart rate was recorded every 10 min.

#### Diet plan

The individual basal metabolic rate was first calculated using the Mifflin equation (Mifflin et al., 1990), and the dietary plan was designed based on the individual basal metabolic rate recorded. During the four-week program, the calorie total of each meal was prescribed, with the percentage values for the intake of high quality proteins, fats, and carbohydrates set as 25–35%, 10–15%, and 55–65%, respectively. The breakfast, lunch, and dinner calorie ratio for the three daily meals was approximately 3:4:3. Nutrients, including vitamins, minerals, essential amino acids, fiber, and polyunsaturated fatty acids, were carefully prescribed for each meal (Wang, Chen & Chen, 2011). A health education course that developed scientific and healthy eating habits was also arranged to enhance the execution of the diet plan.

### Anthropometric measures

BMI, calculated as weight (kg)/height (m) squared, is the primary anthropometric measure. Weight was measured using a digital scale (Yaohua Weighing System Co., Shanghai, China), and height was measured using a wall-mounted stadiometer (TANITA, Tokyo, Japan). Participants in the experiment group were also assessed to determine the waist circumference, which was measured using a flexible ruler, and the percent body fat, which was estimated using an impedance analyzer (TANITA).

### Cognitive function measure: flanker task

The flanker task was employed to assess both basic information processing and executive function, particularly inhibition control. The task was designed and conducted using E-prime 2 software (Psychological Software Tools, Pittsburgh, PA, USA), where two blocks, the practice block and formal block, were included. The stimuli, sized 42% width and 10% height of the visual angle, consisted of five arrows in each block. The stimuli were presented in black color against a white background in the center of a 15-inch LCD screen. Two types of conditions, the congruent condition (i.e., →→→→→ or ←←←←←, 50% probability) and the incongruent condition (i.e., →→← → → or ←← → ←←, 50% probability) were included. In each trial, a black cross was first presented for 500 ms as a cue, followed by the presentation of either the congruent or the incongruent stimuli for 1,500 ms max., and then a blank interval between 200 and 1,000 ms. During the task, participants were instructed to press the response buttons on a keyboard, using their right index fingers, in accordance with the direction of the central target arrow (the number “3” button and number “1” button were used for the right and left directions, respectively) as fast as possible within 1,500 ms. Once a response was made, a blank interval lasting between 200 and 1,000 ms was immediately presented. Failures to respond within 1,500 ms, incorrect responses, and responses made outside of three standard deviations of each participant were counted as incorrect responses. The formal block, with 120 trials, was conducted only after the accuracy in the practice block was higher than 85%. Four behavior indices, the reaction time and the accuracies associated with the congruent and incongruent conditions, were recorded and determined for each participant.

### Statistical analysis

A two-way mixed design was employed. The independent *t*-test and chi-squared test were performed to compare the demographic backgrounds between experiment and control groups, where appropriate. Paired *t*-tests were performed to compare the difference in terms of anthropometric measures between two time points (pre-test vs. post-test) within the group.

A 2 (group: experiment vs. control) × 2 (time point: pre-test vs. post-test) repeated-measure analysis of variance (ANOVA) was separately conducted for the indices from the flanker task with respect to reaction time and response accuracy for the congruent and incongruent conditions. Follow-up analyses using pairwise comparisons with Bonferroni adjustments were applied. All statistical values were reported with Greenhouse–Geisser corrections, and the partial eta-squared (${\mathrm{\eta }}_{p}^{2}$) value is reported.

A Pearson correlation was employed to measure the correlation between the changes of anthropometric measures (i.e., BMI difference between post-test and pre-test in both groups; post- and pre-test difference in waist circumference and in the percent body fat in the experiment group) and the changes in cognitive performances (i.e., the post- and pre-test difference in indices in the flanker task). An alpha value of 0.05, prior to Bonferroni adjustments, was set as the significance level (SPSS 17.0).

## Results

### Demographic variables

Despite the significant difference in the pre-test BMI that was observed between the experiment and the control groups, no other demographic variables in the pre-test reached significant difference between the two groups (*p* > 0.05, Table 1).

### Anthropometric measures

The independent *t*-test revealed significant differences in changes in weight and BMI between the two groups, with a larger reduction in the experimental group than the control group (*p* < 0.01, Table 1).

The paired *t*-test revealed a significantly decreased weight and BMI from post- to pre-test in the experiment group (*p* < 0.01) but not in the control group (*p* > 0.05). The paired *t*-test also revealed a significant difference between post- and pre-test in waist circumference (90.97 ± 9.62 cm vs. 99.70 ± 9.65 cm) and percent body fat (34.77 ± 7.52% vs. 39.40 ± 6.07%) in the experimental group (*ps* < 0.01).

### Flanker task

#### Congruent condition

Regarding the reaction time, the two-way ANOVA revealed a main effect of the time point (*F*(1, 56) = 27.37, *p* < 0.01, ${\mathrm{\eta }}_{\mathrm{p}}^{2}=0.33$), with a shorter time observed for the post-test (368.08 ± 29.15 ms) than the pre-test (388.08 ± 35.35 ms). The analysis also revealed a main effect of the group (*F*(1, 56) = 10.02, *p* < 0.01, ${\mathrm{\eta }}_{\mathrm{p}}^{2}=0.15$), with a shorter time observed for the control group (366.65 ± 28.36 ms) than the experiment group (389.08 ± 31.96 ms). There was no interaction between the time point and the group (*p* > 0.05).

For the accuracy, the analysis revealed no main effect of the time point or the group (*ps* > 0.05) or for the interaction of time point and group (*p* > 0.05).

#### Incongruent condition

For the reaction time, the two-way ANOVA revealed a main effect of the time point (*F*(1, 56) = 36.89, *p* < 0.01, ${\mathrm{\eta }}_{\mathrm{p}}^{2}=0.40$), with a shorter time observed for the post-test (392.51 ± 30.98 ms) than the pre-test (415.69 ± 38.31 ms). The analysis also revealed a main effect of the group (*F*(1, 56) = 8.57, *p* < 0.01, ${\mathrm{\eta }}_{\mathrm{p}}^{2}=0.13$), with a shorter time observed for the control group (392.30 ± 32.09 ms) than the experiment group (415.12 ± 33.28 ms). There was no interaction between the time point and group (*p* > 0.05).

**Figure 2:**
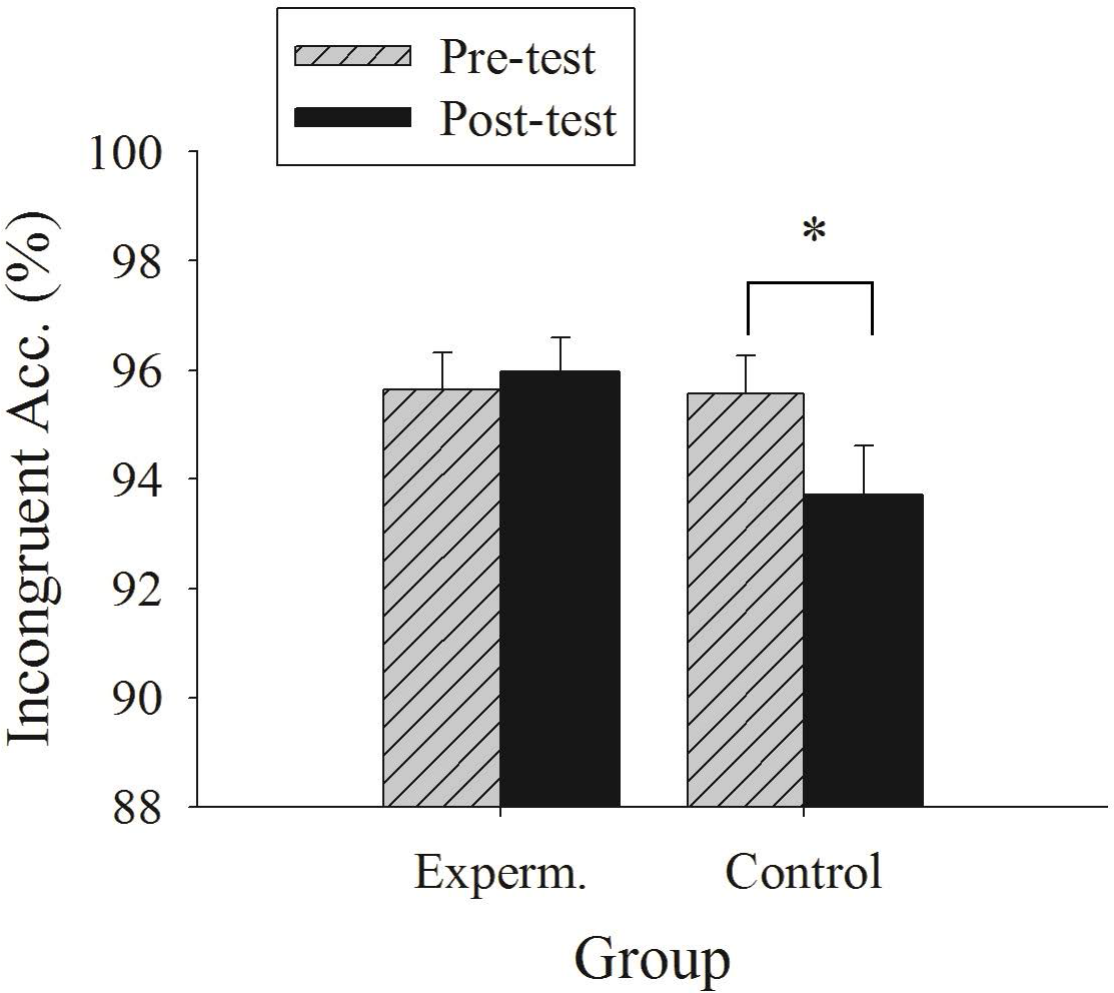
Illustration of the interaction between group and time point in the incongruent accuracy of the flanker task.

For the accuracy, the analysis revealed no main effect of the time point or the group (*ps* > 0.05). However, a significant interaction of the time point and group was observed (*F*(1, 56) = 4.88, *p* < 0.05, ${\mathrm{\eta }}_{\mathrm{p}}^{2}=0.08$). The follow-up multiple comparison revealed a higher accuracy for the experiment group (95.97 ± 3.42%) than the control group (93.71 ± 4.76%) for the post-test; a difference between the experiment and the control group was not observed for the pre-test (*p* > 0.05). Additionally, although there was no significant difference between post-test and pre-test for the experiment group (*p* > 0.05), a lower accuracy for the post-test (93.71 ± 4.76%) than the pre-test (95.57 ± 3.70%) was observed for the control group (Fig. 2).

### Correlation between the changes in anthropometric measures and cognitive function

**Figure 3:**
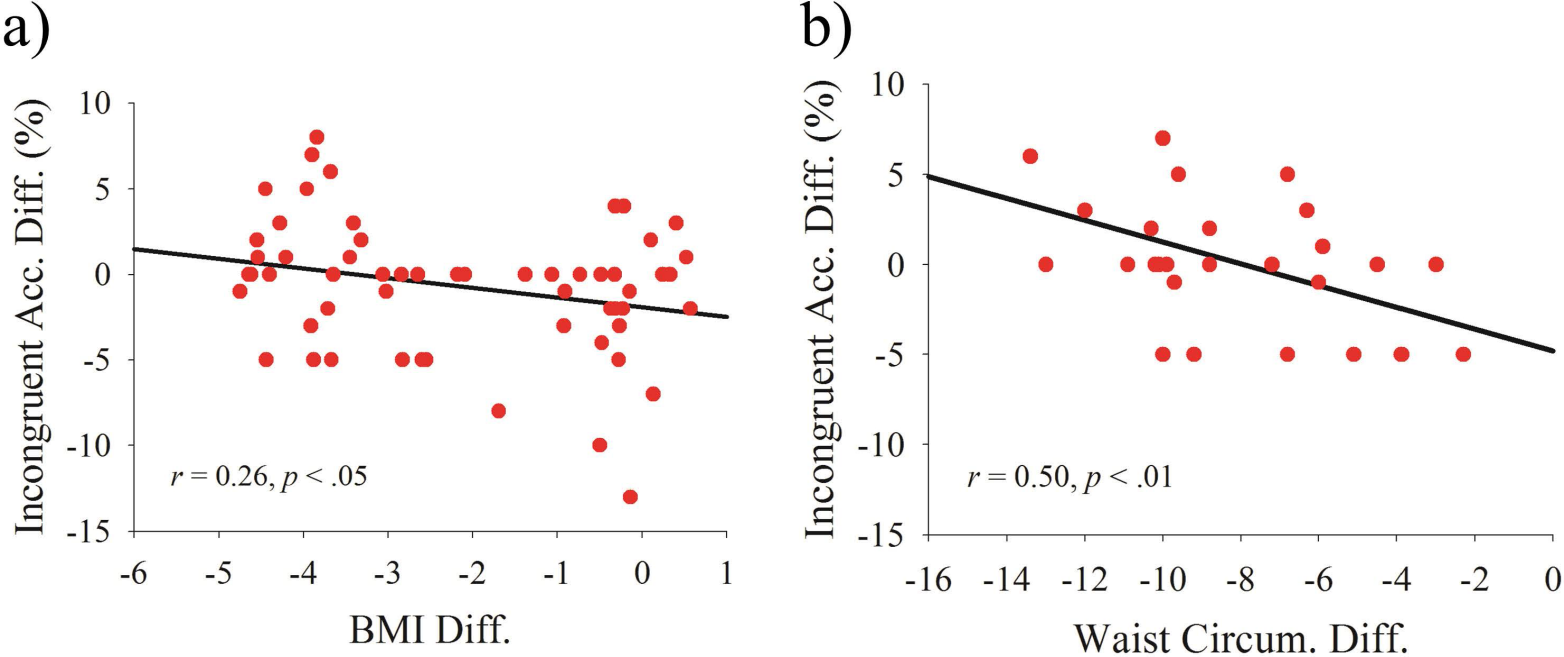
Scatterplots of the relationship between post- and pre-test differences in (A) body mass index and incongruent accuracy and in (B) waist circumference and incongruent accuracy.

The analysis revealed a significant correlation between the difference in BMI and the difference in the incongruent accuracy (*r* =  − 0.26, *p* < 0.05, Fig. 3A), but no other correlations were observed. Similarly, a significant correlation was observed between the difference in waist circumference and the difference in the incongruent accuracy (*r* =  − 0.50, *p* < 0.01, Fig. 3B), and no other correlations were observed. Lastly, no correlations were observed between the difference in the percent body fat and the difference of the cognitive function (Table 2).

**Table 2:** Correlation matrix between changes of anthropometric measures and flanker task

	Congruent RT (−)	Incongruent RT (−)	Congruent ACC (+)	Incongruent ACC (+)
BMI (kg/m^2^) (−)	0.01	−0.04	−0.14	−0.26^*^
Waist circum. (cm) (−)	−0.01	−0.03	−0.12	−0.50^**^
Percent body fat (%) (−)	−0.09	−0.05	−0.16	−0.13

**Notes.**

* *p* < 0.05.

** *p* < 0.01.

(+) the higher value presents better performance (−) the lower value presents better performance Waist circum. Waist circumference RT reaction time (ms) ACC accuracy (%)

## Discussion

The current study was among the first to determine the effects of a combined exercise and dietary program on the cognitive function of obese adolescents, with an additional examination of whether the combined-program-induced weight change was associated with the cognitive function. Our primary results demonstrated that the participants in the combined program not only had reduced BMI but also maintained their cognitive performances in terms of the incongruent accuracy of the flanker task; however, a significant reduction in incongruent accuracy was observed in the control group counterparts following a four-week period. Additionally, the variables associated with reduced weight status were correlated with the better incongruent accuracy.

Previous studies have shown that a combined exercise and dietary program can reduce weight and alter the obesity-related physiological (e.g., BMI, body fat percentage) and metabolic levels (e.g., high-density lipoprotein cholesterol, fasting glucose, and triglyceride) of obese adults (Ho et al., 2013; Smith et al., 2010), and the present study extends these beneficial effects to cognitive function. Although the main effects for time and for group in reaction time were revealed, suggesting that there may be a learning effect and that control group posited better cognitive function than experimental group, the latter interaction effect of group and time in accuracy suggested that the intervention benefits cognitive function and the facilitation was not resulted from speed-accuracy trade off. Notably, the improvement in cognition attributed to the combined program relative to the wait-list control group was exclusively observed for the incongruent but not the congruent condition. The incongruent condition reflects executive function, particularly in terms of inhibition, whereas the congruent condition reflects relative basic information processing (Drollette et al., 2014; Voss et al., 2011). Executive function, recognized as processes that are of higher order and require a higher cognitive demand, is responsible for purposeful and goal-directed behavior (Berger, Pargman & Weinberg, 2002; Hillman et al., 2014). That is, the positive effect of the combined program on cognitive function was disproportionate, where the task condition engaged more of the cognitive demand and had larger effects. This finding may be particularly relevant to the obese population, because obesity has been linked to impaired executive function in several domains (Fitzpatrick, Gilbert & Serpell, 2013; Reinert, Po’e & Barkin, 2013) and because BMI is significantly correlated with brain structure deficits that are associated with executive function (Kamijo et al., 2012; Reinert, Po’e & Barkin, 2013).

Another novelty was to examine how the combined-program-induced weight reduction affected cognitive function. While a negative relationship between obesity status and cognitive function has generally been observed, a majority of studies have employed a cross-sectional design (Fitzpatrick, Gilbert & Serpell, 2013; Reinert, Po’e & Barkin, 2013). This study design compared the difference in cognitive performance between obese and non-obese populations and evaluated the association instead of a causal effect. It should be noted that the relationship among physical activity, diet, cognition, and obesity is rather simple. Chang et al. (2016) indicated that an intervention (i.e., physical activity) and obesity may affect cognitive function through independent or overlapping approaches. Additionally, physical activity or obesity could also act as either a moderator or mediator to influence cognition. Our findings showing that the weight changes from the intervention were positively correlated with changes in cognitive function, from a longitudinally designed perspective, imply that weight status may mediate the effects of the combined program on cognitive function. This viewpoint not only provides further interpretation of the priority sequence for these variables but also highlights the importance of weight loss for cognitive function in this specific population.

The beneficial effect related to the combined physical activity and dietary program on cognitive function may be attributed to physical activity, dietary restriction, or interaction of both factors. For example, parasympathetic alteration resulting from a short-term physical activity program has been linked to increased executive function in obese adolescents, suggesting a role of cardiac autonomic control in the connection between exercise and cognitive function (Chen et al., 2016). Brain mechanisms underlying the effects of the physical activity and executive function have also been proposed. Following an afterschool physical activity program, children showed both better performance in executive function tasks and larger brain activation in terms of the P3 component of electrophysiological measures, suggesting that physical activity is associated with increased attentional resources to the given task (Chang et al., 2013; Hillman et al., 2014). Functional magnetic resonance imaging (fMRI) also linked the physical activity intervention to modulation of the neural circuitry of executive function, such as increased activation in the frontal gyrus and anterior cingulate (Krafft et al., 2014). Interestingly, lower activation within these cortical networks was observed in the obese population, highlighting the essential role of physical activity in executive function, brain, and obesity. Although the relationship between diet and the brain is less understood, both dairy and vegetable intake were inversely related to brain iron concentrations in deep gray-matter structures (Hagemeier et al., 2015). The results are worth consideration because an increased iron load in specific brain regions (e.g., hippocampus and hypothalamus) has been observed in obese people, and the higher brain iron overload, along with obesity-induced insulin resistance, may contribute to the poorer cognitive performance (Blasco et al., 2014). Lastly, both exercise and diet may also work together to affect cognition. Animal studies have demonstrated that voluntary wheel running and caloric restriction increased the dendritic spine density and brain-derived neurotrophic factor (BDNF) level in the hippocampus of diabetic mice, suggesting that increased energy expenditure and attenuated energy intake contribute to hippocampal plasticity (Stranahan et al., 2009).

The major strengths of the study include the examination of a combined program for weight reduction and multiple aspects of cognitive function, as well as the study of the correlation between weight reduction and the cognitive function among obese adolescents. However, our quasi-experimental design, with its relatively small sample size and short-term intervention, was of concern since these factors could result in a lack of causal effect. Future studies that employ a randomized controlled trial with larger scale and longer duration of intervention are needed to replicate the findings. Additionally, previous studies have found certain confounding variables associated with cognitive function, such as demographic background (social economic status and parental education), fitness level, and even time spent playing video games and in front of screens. Future studies should take these variables into account to strengthen the causal effect. Nonetheless, our findings warrant further investigation. Furthermore, the combined program positively and disproportionately affected tasks related to executive function; nevertheless, the incongruent condition of the flanker task mainly reflects the inhibition aspect of executive function. Rather than a union construct, several distinguishable sub-components have been identified within executive function, such as inhibition, sifting, and updating (Miyake et al., 2000), as well planning (Chang et al., 2012). Generalization in terms of our program’s effects on executive function should therefore proceed cautiously. Lastly, the lack of diet choice based on personal traits may be another limitation. Although the dietary plan was carefully tailored, the percentage and amount of food components were our primary concern. Given that both personal and behavioral attributes can affect eating behavior (Dressler & Smith, 2013), these factors are worth future consideration.

## Conclusion

The combined four-week exercise and dietary program may benefit cognitive function, with a disproportionately larger influence on executive function, in adolescents with obesity. The beneficial effect on executive function attributed to the combined program may be associated with the extent of weight reduction, suggesting that weight may connect the intervention and the effects on executive function.

## Supplemental Information

Data S1 Raw data

### Additional Information and Declarations

#### Data Availability

The following information was supplied regarding data availability: The raw data has been supplied as a Supplementary File.
